# Determination of Protein Interaction in Milk Protein Concentrate Powders Manufactured from pH-Adjusted and Heat-Treated Skim Milk

**DOI:** 10.3390/foods13233832

**Published:** 2024-11-28

**Authors:** Kavya Dileep, Hari Meletharayil, Jayendra K. Amamcharla

**Affiliations:** 1 Department of Animal Sciences and Industry, Food Science Institute, Kansas State University, Manhattan, KS 66506, USA; kavyad@ksu.edu; 2 Dairy Management Inc., Rosemont, IL 60018, USA; hari.meletharayil@dairy.org; 3 Midwest Dairy Foods Research Center, Department of Food Science and Nutrition, University of Minnesota, St. Paul, MN 55108, USA

**Keywords:** MPC powders, heating, pH, whey protein, caseins, physicochemical properties

## Abstract

The influence of heating as a pretreatment on the structural and functional attributes of milk protein concentrate (MPC) powders derived from ultrafiltered/diafiltered (UF/DF) skim milk is under-reported. This research delves into the impact of pH and heat treatment on skim milk’s properties before UF/DF and how these changes affect the resulting MPC powders. By adjusting the pH of skim milk to 6.5, 6.8, or 7.1 and applying thermal treatment at 90 °C for 15 min to one of two divided lots (with the other serving as a control), we studied the protein interactions in MPC. Post-heat treatment, the skim milk’s pH was adjusted back to 6.8, followed by ultrafiltration and spray drying to produce MPC powders with protein content of 82.38 ± 2.72% on a dry matter basis. MPC dispersions from these powders at 5% protein (*w*/*w*) were also evaluated for particle size, viscosity, and heat coagulation time (HCT) to further understand how the protein interactions in skim milk influence the properties of MPC dispersions. Capillary electrophoresis was used to assess the casein and whey protein distribution in both the soluble and colloidal phases. Findings revealed that preheating skim milk at pH 7.1 increased serum phase interactions, while heating skim milk preadjusted to a pH of 6.5 promoted whey protein–casein interactions at the micellar interface. Notably, the D (4,3) of casein micelles was larger for dispersions from milk with a preheated pH of 6.5 compared to other pH levels, correlating positively with enhanced dispersion viscosity due to increased volume fraction. These results support the potential for tailoring MPC powder functionality in various food applications through the precise control of the milk’s pre-treatment conditions.

## 1. Introduction

Increasing demand for food proteins can be observed in the consumer market due to the world’s population growth and changing lifestyles. This market expansion is driven by increased health awareness among consumers due to lifestyle-related diseases and conditions. In addition to age-related muscle loss, the recent rise in the usage of anti-obesity medications also causes muscle loss as a side effect, warranting additional protein intake through diet. A huge part of this rising demand for high-quality proteins is met by milk protein concentrate/isolates and whey protein concentrates/isolates in a wide range of food formulations such as high-protein beverages and nutritional bars.

Milk protein concentrate (MPC) powder is an important milk-derived ingredient that provides high-quality milk protein in the same ratio as in milk with added benefits of excellent nutritional and functional properties in a variety of food applications [1] MPCs are manufactured widely using the ultrafiltration (UF)/diafiltration (DF) process. Membrane technologies provide numerous benefits over traditional methods, such as low-temperature operation, no phase transition, high separation efficiency, and high permeate flux productivity. Additionally, from an industrial perspective, they offer low energy usage, simple equipment, easy operation, and straightforward scalability [2]. The technology uses polymeric or ceramic membranes with specific pore sizes to fractionate whey proteins and caseins from lower-molecular-weight components such as lactose and minerals [3]. Skim milk is concentrated to varying volumetric concentration factors depending on the amount of lactose and minerals that need to be removed with respect to the desired final protein content. As the concentration factor increases, the viscosity of the retentate tends to increase. In such cases, for efficient and further removal of lower-molecular-weight compounds and to manage viscosity, a process known as diafiltration (DF) is used wherein a diluent (generally water) is added to the concentrated retentate. The UF/DF retentate can be further spray dried to manufacture MPC powders varying in protein content (35–90% *w*/*w*) depending upon the concentration factor achieved.

Thermal treatment is an important processing step in the manufacture of dairy products. Various factors like ionic strength, ionic composition, protein concentration, and pH affect heat treatment’s impact on the final product’s physicochemical properties [4,5]. The heat treatment of proteins leads to the denaturation or aggregation of proteins. At temperatures greater than 70 °C, an irreversible unfolding of whey proteins occurs, which causes them to denature and expose reactive functional groups such as the free thiol groups in β-lactoglobulin (β-LG). These functional groups can thereafter aggregate through hydrophobic bonding and thiol/disulphide exchanges with corresponding denatured whey proteins, caseins (CN), or κ-caseins (κ-CN), resulting in the formation of whey protein aggregates, whey protein–CN aggregates, or whey protein–κ-CN complexes [6,7,8]. Numerous studies on skim milk have investigated the protein interactions due to heating and have associated pH at heating to be a major contributing factor in the distribution of proteins and type of interactions. Denatured whey proteins were found to form filamentous appendages at the casein micelle surface upon heating at higher temperatures and at pH values below 6.7, whereas serum-phase whey protein aggregates were found at higher pH values [9,10]. In a study of heated skim milk systems at a temperature of 80–90 °C for about 600 s [11], the association of whey proteins with casein micelles at lower pH values was determined to be greater than 80% while only about 20% were found when skim milk was heated at pH > 6.8.

In the manufacture of MPC powders, pasteurization is typically employed as a heat kill step. The denaturation of whey proteins is minimal at pasteurization temperatures. When MPC powders are dispersed for the manufacture of high-protein nutritional beverages that are typically exposed to UHT/retort temperatures, the kinetics of whey protein denaturation and interaction can be different due to differences in ionic strength and calcium ion activity in the formulation when compared to skim milk. Heating these MPC dispersions at different preheating pH values yields a heat coagulation time/pH curve different from skim milk [1]. Studies [12,13] showed that the pH adjustment and heating of liquid MPC had significant effects on the viscosity, Ca^2^⁺ activity, and heat stability of the concentrates. Another study demonstrated that MPC powders manufactured from liquid ultrafiltration concentrate heat treated at ≥100 °C proved challenging to disperse and solubilize [14]. The earliest study on the preheating treatment of skim milk during the manufacture of MPC powders was carried out by Carr [15] on MPC85 powders to characterize the rheology of the concentrate and functional properties of the powder. Lin et al. [16] manufactured MPC powders from skim milk that was heat treated at 72 °C for 15 s (LHMPC) or 85 °C for 30 s (MHMPC) and studied the functionality of the corresponding reconstituted MPC dispersions. Meena et al. [17] investigated the effect of a change in the pH of skim milk prior to ultrafiltration on the final MPC70 powders and concluded that there were significant changes in the functional properties of the treated skim milk.

The existing research underscores the finding that both the thermal processing and pH modulation of skim milk before the production of milk protein concentrate (MPC) powders can markedly influence the functional characteristics of the finished product. However, there is a research gap concerning the impact of skim milk preheated and pH adjusted to different levels on the functional properties of MPC dispersions. The current study addresses this gap by examining how the pH adjustment and thermal treatment of skim milk prior to ultrafiltration/diafiltration (UF/DF) can be optimized to modify protein interactions. Such adjustments have the potential to alter the physicochemical and functional properties of both MPC powders and their subsequent dispersions, offering valuable insights for the enhancement of food systems incorporating MPC.

## 2. Materials and Methods

### 2.1. Manufacture of Functionally Modified MPC Powders

#### 2.1.1. Milk Supply and Treatment

Fresh milk was procured from South Dakota State University Dairy Farm (Brookings, SD, USA). The freshly obtained milk (3500 lbs) was separated using a cream separator (model 392, Indianapolis, IN, USA), and skim milk was pasteurized at 71.7 °C for a hold time of 15 s. Skim milk, with an initial pH of 6.7, was then divided into three equal parts, each amounting to 1100 lbs, and their pH values were adjusted to 6.8 (~100 mL NaOH), 6.5 (~900 mL HCl), and 7.1 (~18,000 mL NaOH), respectively, using 1 N HCl or 0.5 N NaOH at room temperature. Distilled water was used to maintain the dilution constant for all the treatments. Each pH-adjusted sample was further divided into equal halves of 550 lbs. One half was left unheated, while the other half was heat treated at 90 °C for 15 min. Both the heated (subjected to a post-pasteurization heat treatment) and unheated (not subjected to a post-pasteurization heat treatment) samples were readjusted to pH 6.8 and cooled to <4 °C for further processing.

#### 2.1.2. Ultrafiltration of Heat-Treated Skim Milk

Ultrafiltration (UF) was performed on each treated milk sample using two parallelly arranged 10 kDa polyether sulfone spiral wound membranes (3838 element format, Dominick Hunter Filtration Division—N.A, Parker Hannifin Corporation, Oxnard, CA, USA). Each membrane element had a diameter of 97 mm and length of 965 mm with a 1.1 mm feed spacer and a total surface area of 5.7 m^2^. Ultrafiltration was performed at 15° C. The inlet and outlet pressures were maintained at 50 and 20 psi, respectively, resulting in a transmembrane pressure of 30 psi. Skim milk was concentrated to a volumetric concentration factor of 5×. To achieve a protein content of 80–85% while effectively reducing lactose and mineral content in the final milk protein concentrate, 65–75% diafiltration water was utilized.

#### 2.1.3. Spray Drying

**Figure 1:**
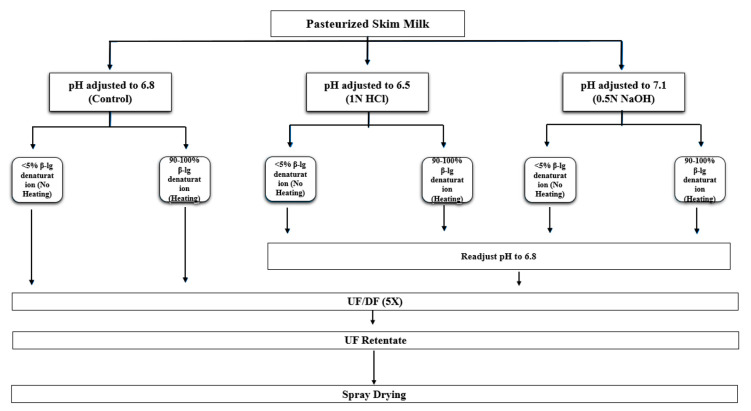
Experimental design for the manufacture of MPC powders heated and unheated at different pH values.

The UF retentates were spray dried in a single-stage pilot-scale dryer fitted with a Niro centrifugal disk atomizer (ASO 412E, Niro Inc., Columbia, MD, USA). The inlet and outlet temperatures were maintained at 185 °C and 105 °C, respectively. The powders were packed in airtight containers and stored at −21 °C until further analysis. The complete experimental design is provided in Figure 1.

The composition of pasteurized skim milk (fat, lactose, and total solids) was obtained using DairySpec FT (Bentley Instruments Inc., Chaska, MN, USA). The total protein, Non-Casein Nitrogen (%), and Non-Protein Nitrogen (%) were analyzed using the standard Kjeldahl methods [18] with a multiplication factor of 6.38.

### 2.2. Analysis of Protein Interaction in the Functionally Modified MPC Powders

Separation of serum phase to determine soluble-phase serum proteins: The serum phase was separated from MPC powder reconstituted to 5% (wt./wt.) protein dispersion using the method described by Lucey [19]. MPC dispersions were ultracentrifuged at 90,000× *g* at 20 °C for 60 min to determine soluble-phase whey proteins and their association with casein micelles. The carefully collected supernatant was used for capillary gel electrophoresis. The difference in the amounts of β-lactoglobulin (β-lg) and α-lactalbumin (α-lac) in the ultracentrifuged supernatant of unheated and heated MPC dispersions was determined as the percentage of whey proteins associated with casein micelles [19].

Capillary gel electrophoresis (CGE): Supernatants from ultracentrifuged MPC dispersions were analyzed using CGE to determine individual protein fractions. A Beckman P/ACE MDQ capillary electrophoresis system (Beckman-Coulter, Fullerton, CA, USA) equipped with a UV detector set at 214 nm was used to carry out CGE. The separation was performed using a 50 μm bare fused silica capillary (20.2 cm effective length from the inlet to the detection window) following the method suggested by Salunke et al. [20]. To estimate the molecular weight of proteins in each sample, an SDS-MW size standard (recombinant proteins 10–225 kDa supplied with the ProteomeLab SDS-MW Analysis Kit) was used. A capillary preconditioning method was performed after every three samples, involving a basic rinse (0.1 N NaOH, 5 min, 344.7 kPa), an acidic rinse (0.1 N HCl, 2 min, 344.7 kPa), a water rinse (HPLC grade water, 2 min, 344.7 kPa), and an SDS Gel rinse (SDS gel fill, 10 min, 275.8 kPa). Following these preconditioning steps, the sample was electrokinetically introduced at 5 kV for 20 s. The separation process was conducted at a constant voltage of 15 kV (25 °C and 20 bar pressure) with reverse polarity in the SDS-molecular-weight gel buffer (Salunke et al. [20]). The peaks in the capillary electropherogram were identified and compared. Using a valley-to-valley approach, the area of each identified individual CN fraction (αS1-CN, αS2-CN, β-CN, κ-CN, and γ-CN), serum protein fraction (α-lac, β-lg, peptides (peaks between 10 kDa and 20 kDa)), and NPN fraction (all positive peaks below 10 kDa) was calculated as percentage of total area (positive peaks).

### 2.3. Characterization of Heat-Induced Changes in MPC Dispersions from MPC Powders

#### 2.3.1. Mean Particle Size and Zeta Potential

Mean particle size and zeta potential were analyzed using a dynamic light scattering analyzer (DelsaMax Assist, Beckman Coulter, CA, USA). Samples were prepared for measurement by diluting to 1/100 with distilled water. Disposable cuvettes were used for the analysis of particle size, and for zeta potential, samples were injected into the flow cell using a syringe at room temperature.

#### 2.3.2. Apparent Viscosity

MPC powders were reconstituted to a protein concentration of 5% *w*/*w* in distilled water at 45 °C under continuous stirring using a magnetic stirrer for 30 min and kept overnight in a refrigerator for complete rehydration. The apparent viscosity was measured at a varied shear rate between 10 s^−1^ to 300 s^−1^ at 25 °C using a stress–strain-controlled rheometer (MCR-92 Anton Paar, Vernon Hills, IL, USA) fitted with a 50 mm diameter stainless steel cone with a 1° angle and a 101 μm gap [21]. Using the power law model, the consistency coefficient and flow behavior index were also calculated.

$$\sigma =K\;{\dot{\gamma }}^{\eta }$$

where *σ* = shear stress, $\dot{\gamma }$ = shear rate, *K* = consistency coefficient, and *η* = flow behavior index.

#### 2.3.3. Heat Coagulation Time

MPC powders reconstituted to a 5% *w*/*w* protein concentration as described above were used for measuring the heat coagulation time. Heat-resistant screw-cap test tubes (8 mL, 17 mm × 63 mm; DWK Life Science, Millville, NJ, USA) containing three milliliters of MPC dispersions were inserted on a stainless-steel rack. The rack with the tubes was immersed in an oil bath (Narang Scientific Works Pvt. Ltd., New Delhi, India) maintained at 140 °C and placed on a rocker. The time was recorded from the moment the tubes were immersed in the oil bath until the onset of coagulation was observed in the samples. This time was noted as the heat coagulation time [12,22].

### 2.4. Statistical Analysis

Statistical analysis was performed using PROC GLIMMIX and Tukey’s test in SAS Version 9.4 (SAS Institute Inc., Cary, NC, USA) to determine significant differences based on treatments. The significance was established when *p* < 0.05.

## 3. Results and Discussion

The composition of skim milk that was used as feed to make the individual lots of MPC powders was fat—0.07%; protein—3.31%; total protein—3.51%; lactose—4.88%; total solids—9.27%; SNF—9.19%. The mean protein contents of MPC powders from heated and unheated skim milk were not significantly different. All other proximate analyses of the samples were not significantly different (Table 1).

### 3.1. Analysis of Protein Interaction in MPC Dispersions from MPC Powders

**Figure 2:**
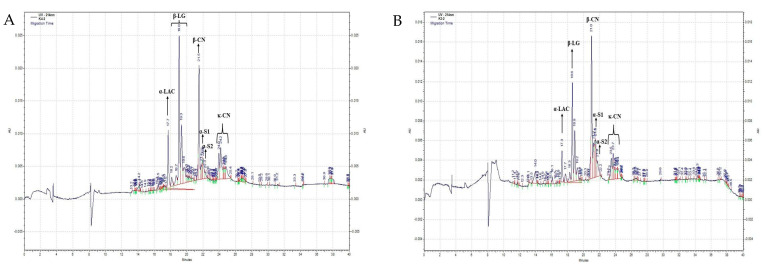
Typical capillary gel electropherogram of serum phase of (**A**) MPC dispersions of MPC powders manufactured from unheated skim milk at pH 6.8, (**B**) MPC dispersions of MPC powders manufactured from heated skim milk at pH 6.8.

The supernatant obtained after the centrifugation of MPC dispersions was analyzed using capillary electrophoresis to quantify the level of soluble-phase whey protein in the samples. Figure 2A,B provide typical electropherograms obtained for unheated MPC samples and heated MPC samples at pH 6.8, respectively. Table 2 shows the percentages of whey proteins present as a function of the pH in the serum phase of MPC dispersions.

As the pH of preheated skim milk increased from 6.5 to 7.1, an increase in the soluble-phase whey proteins of MPC dispersions was observed. The findings were similar to what was observed by other researchers for skim milk. A majority of the whey proteins (β-lg and α-La) were found to be associated with casein micelles at a preheating pH of 6.5, among which, β-lg (>90%) was the major associated whey protein. With an increase in preheating pH from 6.5 to 7.1, β-lg’s association with casein decreased from ~90% to ~16.6% (*p* < 0.05) in the heated skim milk base. Qi et al. [23] also reported a similar decrease in whey protein association with casein micelles when skim milk was preheated at 7.1. Crowley et al. [24] reported that <50% of B-lg is present in the supernatant of heated MPC dispersions at pH 7.1. This is lower than what we observed in our study, and this can be correlated to an increased serum-phase association of whey protein and casein in skim milk systems than in MPC dispersions, owing to the difference in the serum-phase compositions of the two systems, including Ca^2+^ ion activity.

#### 3.1.1. Mean Particle Size and Zeta Potential

The mean particle size of the dispersions increased as the preheating pH decreased from pH 7.1 to 6.5. Heated dispersions across the pH range had a higher mean particle diameter greater than the unheated dispersions (Table 3). Dispersions preheated at pH 6.5 had the higher particle size when compared to all other heated dispersions. The particle size of the heated dispersions was also positively correlated with the viscosity of the dispersions. Increased micellar phase interactions (whey protein and casein interactions) can explain the increase in the particle size of dispersions preheated at pH 6.5. Heat treatment at pH 6.5 resulted in an increased association of whey proteins with the casein micelles, resulting in a larger mean particle size. Greater serum-phase interactions of dispersions preheated at pH 7.1 can explain the minimal increase in particle size. The zeta potential values of both heated and unheated MPC dispersions were not statistically significant (*p* > 0.05) at any pH considered in this study.

#### 3.1.2. Apparent Viscosity

The apparent viscosity of dispersions at pH 6.5, 6.8, and 7.1 for both unheated and heated samples is provided in Table 4. Due to volume fraction and casein micelle voluminosity depending on the pH and heat treatment, viscosity changes are expected during thermal processing [25]. In the current study, the viscosity of heated dispersions was found to be higher than the viscosity of unheated dispersions. The apparent viscosity of MPC dispersions preheated at a pH of 6.5 was the highest when compared to MPC dispersions from all other preheating pH values in the study. This can be attributed to an increase in micellar-phase interactions and an increase in volume fraction [26]. No statistically significant increase in viscosity between MPC dispersions preheated at pH 6.8 and 7.1 and MPC dispersions that were unheated was observed. No significant change in particle size on heating at this preheating pH can explain the minimal increase in the viscosity of the dispersions.

#### 3.1.3. Heat Coagulation Time (HCT)

The heat stability of the MPC dispersions made from unheated and heated skim milk at varying pH values is given in Table 5. The unheated samples at all pH values showed no significant difference (*p* > 0.05) in their heat coagulation time. This led to the conclusion that pH changes alone did not influence the heat stability of the final product. But the heated samples showed a different pattern. While no significant difference (*p* > 0.05) was observed among unheated and heated samples at pH 6.8, a statistically significant (*p* < 0.05) increase was observed in the heated samples at pH 6.5 and 7.1.

Skim milk and MPC dispersions tend to diverge in their heat stability pH curves. In skim milk, a maximum and minimum in heat coagulation times is primarily influenced by pH of heating skim milk. In MPC dispersions, although the maximum and minimum of heat coagulation time is influenced by the pH of preheating the dispersions, the solvent quality of the MPC dispersions has a major role to play in HCT. Crowley et al. [23] showed that the protein content of MPC powders used for MPC dispersions can have an influence on HCT. This was associated with differences in calcium ion activity and ionic strength. Sunkesula et al. [1] reported an increase in the HCT of MPC dispersions preheated at pH 6.8 and a decrease in the HCT of MPC dispersions preheated at pH 7.1. This was also corelated with a difference in calcium ion activity at this preheating pH. A difference in calcium ion activity can influence the interactions in caseins and whey proteins during thermal treatments, resulting in differences in HCT.

In the current study, the HCT of MPC dispersions had a maximum value at a preheating pH of 7.1 and 6.5. Since the preheating was carried out in skim milk before the manufacture of MPC dispersions, protein interactions occurred at the ionic strength and calcium ion activity of skim milk. The distribution of casein and whey protein interactions will mimic interactions happening in skim milk. Therefore, HCT will not be influenced by the solvent quality (ionic strength) of MPC dispersions, and one can observe the predictable behavior of the HCT of MPC dispersions when preheating is carried out in skim milk.

## 4. Conclusions

This study illustrated that the combination of heat treatment and minor pH adjustments on skim milk base before manufacturing high-protein MPC powders can influence the physicochemical properties of the resulting powders. A high heat treatment on skim milk at 90 °C for 15 min at pH 6.5, 6.8, and 7.1 resulted in protein–protein interactions. These results were similar to the previous literature available on skim milk alone. In the resulting MPC powders, a greater amount of unbound whey proteins was found in samples with a heated skim milk base at pH 7.1, while most whey proteins were found to be bound to casein micelles in samples with a heated skim milk base at pH 6.5. These results indicate that while heating did cause the dissociation of caseins from the micelles, increasing the pH to 7.1 resulted in more stable casein micelles in the colloidal phase and more whey protein aggregate formations in the serum phase. Differences in pH during heating therefore affected the physicochemical properties, mainly viscosity, which showed a significant increase in samples with a heated skim milk base at pH 6.5. Heat stability was observed to have improved in samples with the pH adjusted to 6.5 and 7.1. The findings of the current research can be helpful in final product applications such as coffee/tea beverages or baked foods that require high-temperature processing or products like yogurt or cheese where the viscosity of the final product is a key quality parameter. Further studies on the change in viscosity and heat stability by using acid gelation can help gain further understanding of the potential of the functionally modified MPC powder in new or improved product applications.

## Figures and Tables

**Table 1:** Protein and total solids (TS) content (%) of MPC powders manufactured from unheated and heated skim milk.

Attribute	pH 6.5		pH 6.8		pH 7.1	
	Unheated	Heated	Unheated	Heated	Unheated	Heated
Total Protein (%)	83.15 ± 1.12	81.08 ± 1.71	82.72 ± 0.20	81.95 ± 0.67	83.29 ± 0.43	82.09 ± 1.36
Non-Casein Nitrogen (%)	9.12 ± 5.56	4.42 ± 0.17	11.03 ± 3.63	5.39 ± 0.24	11.43 ± 3.09	4.40 ± 0.21
Non-Protein Nitrogen (%)	1.37 ± 0.39	1.89 ± 0.59	1.98 ± 1.03	1.30 ± 0.35	1.26 ± 0.21	1.50 ± 0.29
Total Solids (%)	97.45 ± 0.72	96.36 ± 0.53	97.74 ± 0.31	97.45 ± 0.31	97.16 ± 0.70	96.63 ± 0.82

All values are expressed as mean ± SD (*n* = 3).

**Table 2:** Whey protein distribution (%) in the serum phase and colloidal phase after heating for 90 °C for 15 min at heating pH of 6.5, 6.8, and 7.1.

Concentration (%)	Phase	Heating pH		
		6.5	6.8	7.1
**β-Lactoglobulin**	Soluble	9.11	64.29	83.43
	Colloidal *	90.89	35.71	16.57
**α-Lactalbumin**	Soluble	13.83	74.19	44.59
	Colloidal *	86.17	25.81	55.41

* Calculated from duplication samples and reported as the ratio of difference in protein concentration before and after heating to the initial concentration of the same protein.

**Table 3:** Particle size (nm) and zeta potential (mV) of dispersions of MPC powders (5% *w*/*w* protein) manufactured from unheated and heated skim milk at varying pH values.

Attribute	pH 6.5		pH 6.8		pH 7.1	
	Unheated	Heated	Unheated	Heated	Unheated	Heated
**D[4,3] (nm)**	184.12 ± 18.08 ^b^	247.70 ± 32.62 ^a^	181.97 ± 3.48 ^b^	199.17 ± 6.78 ^ab^	173.40 ± 9.90 ^b^	181.85 ± 23.41 ^b^
**D10 (nm)**	53.67 ± 7.26 ^a^	65.92 ± 7.76 ^a^	59.38 ± 5.01 ^a^	57.47 ± 9.34 ^a^	55.54 ± 3.75 ^a^	58.99 ± 3.17 ^a^
**D50 (nm)**	82.54 ± 12.70 ^a^	110.82 ± 27.30 ^a^	89.43 ± 5.47 ^a^	95.91 ± 11.49 ^a^	84.87 ± 8.25 ^a^	89.68 ± 8.69 ^a^
**D90 (nm)**	127.17 ± 39.83 ^a^	212.04 ± 52.35 ^a^	158.00 ± 11.33 ^a^	187.90 ± 45.80 ^a^	140.11 ± 12.31 ^a^	139.95 ± 24.73 ^a^
**Zeta Potential (mV)**	24.27 ± 2.50 ^a^	24.10 ± 2.17 ^a^	23.59 ± 3.02 ^a^	23.29 ± 3.76 ^a^	26.73 ± 1.40 ^a^	26.32 ± 0.92 ^a^

^a,b^ Means within the same row with different superscripts are significantly different (*p* < 0.05). D[4,3] is the volume moment mean diameter. D10, D50, and D90 are the diameters (nm), where 10%, 50%, and 90% of all powder particles have smaller sizes, respectively. All values are expressed as mean ± SD (n = 3).

**Table 4:** Apparent viscosity, flow behavior index (n) and consistency coefficient (K) of MPC dispersions (5% *w*/*w* protein) manufactured from unheated and heated skim milk at varying pH values.

Attribute	pH 6.5		pH 6.8		pH 7.1	
	Unheated	Heated	Unheated	Heated	Unheated	Heated
**Viscosity at 112 mPa·s**	2.32 ± 0.95 ^b^	4.82 ± 0.90 ^a^	2.34 ± 0.16 ^b^	3.29 ± 0.43 ^b^	2.13 ± 0.21 ^b^	2.97 ± 0.63 ^b^
**Flow behavior index, n**	0.95 ± 0.02 ^a^	0.82 ± 0.12 ^a^	0.92 ± 0.05 ^a^	0.81 ± 0.23 ^a^	0.93 ± 0.02 ^a^	0.92 ± 0.05 ^a^
**Consistency index, K (mPa·s)**	0.0030 ± 0.00 ^a^	0.0145 ± 0.01 ^a^	0.0037 ± 0.00 ^a^	0.0145 ± 0.01 ^a^	0.0030 ± 0.00 ^a^	0.0046 ± 0.00 ^a^

^a,b^ Means within a row with different superscripts are significantly different (*p* < 0.05). All values are expressed as mean ± SD (n = 3).

**Table 5:** Heat coagulation time (min) of MPC dispersions at 5% *w*/*w* protein manufactured from unheated and heated skim milk at pH 6.5, 6.8, and 7.1.

Heat Coagulation Time (s)		
pH	Unheated	Heated
**6.5**	5.06 ± 0.05 ^b^	14.32 ± 0.09 ^a^
**6.8**	6.10 ± 0.02 ^b^	7.26 ± 0.02 ^b^
**7.1**	4.86 ± 0.01 ^b^	14.20 ± 0.06 ^a^

^a,b^ Means within a row with different superscripts are significantly different (*p* < 0.05). All values are expressed as mean ± SD (n = 3).

## Data Availability

The original contributions presented in the study are included in the article, further inquiries can be directed to the corresponding author.
